# The Role of Gut Microbiota in Neuromyelitis Optica Spectrum Disorder

**DOI:** 10.3390/ijms25063179

**Published:** 2024-03-09

**Authors:** Shi-Qi Yao, Xiayin Yang, Ling-Ping Cen, Shaoying Tan

**Affiliations:** 1 School of Optometry, The Hong Kong Polytechnic University, Hong Kong SAR, China; shiqi.yao@connect.polyu.hk (S.-Q.Y.); xia-yin.yang@connect.polyu.hk (X.Y.); 2 Joint Shantou International Eye Center of Shantou University and The Chinese University of Hong Kong, Shantou 515041, China; cenlp@hotmail.com; 3 Shantou University Medical College, Shantou 515041, China; 4 Research Centre for SHARP Vision (RCSV), The Hong Kong Polytechnic University, Hong Kong SAR, China; 5 Center for Eye and Vision Research (CEVR), 17W Hong Kong Science Park, Hong Kong SAR, China

**Keywords:** neuromyelitis optica spectrum disorders, gut microbiota, metabolites

## Abstract

Neuromyelitis optica spectrum disorder (NMOSD) is a rare, disabling inflammatory disease of the central nervous system (CNS). Aquaporin-4 (AQP4)-specific T cells play a key role in the pathogenesis of NMOSD. In addition to immune factors, T cells recognizing the AQP4 epitope showed cross-reactivity with homologous peptide sequences in *C. perfringens* proteins, suggesting that the gut microbiota plays an integral role in the pathogenicity of NMOSD. In this review, we summarize research on the involvement of the gut microbiota in the pathophysiology of NMOSD and its possible pathogenic mechanisms. Among them, *Clostridium perfringens* and *Streptococcus* have been confirmed to play a role by multiple studies. Based on this evidence, metabolites produced by gut microbes, such as short-chain fatty acids (SCFAs), tryptophan (Trp), and bile acid (BA) metabolites, have also been found to affect immune cell metabolism. Therefore, the role of the gut microbiota in the pathophysiology of NMOSD is very important. Alterations in the composition of the gut microbiota can lead to pathological changes and alter the formation of microbiota-derived components and metabolites. It can serve as a biomarker for disease onset and progression and as a potential disease-modifying therapy.

## 1. Introduction

Neuromyelitis optica spectrum disorder (NMOSD) is a chronic inflammatory autoimmune disease of the central nervous system (CNS). The core clinical manifestations of NMOSD include optic neuritis (ON), acute myelitis, area postrema syndrome, acute brainstem syndrome, and symptomatic cerebral syndrome [1]. In addition to severe visual impairments, people with NMOSD can develop movement disorders and even death [2]. The prevalence of NMOSD ranges from 0.5 to 4 affected per 100,000 with a 9:1 ratio of females to males [3].

The discovery of immunoglobulin G (IgG) antibodies against the aquaporin 4 (AQP4) water channel in patients with NMOSD has been key to the diagnosis of NMOSD [4]. AQP4 is the most widely expressed bidirectional, osmotically driven water channel in the brain, spinal cord, and optic nerves [5]. Within the brain, AQP4 is located in areas of contact with cerebrospinal fluid, localized to the peduncles of astrocytes at the blood–brain barrier (BBB) [6]. Although AQP4 was identified as the primary target, the initial pathophysiologic events that lead to the development of NMOSD remain elusive. In addition to genetic factors, environmental triggers such as plants, bacteria, or viruses may also trigger AQP4-antibodies (Ab) [7,8]. In addition, studies have shown that T cells recognizing AQP4 epitopes are cross-reactive with homologous peptide sequences of commensal bacteria found in the human gut microbiota [9]. Therefore, the influence of the gut microbiota has also been widely studied in recent years as a new risk factor.

The gut microbiota contains about 1200 species of bacteria, and it also includes other organisms such as archaea, protozoa, fungi, and viruses [10], of which Gram-negative Bacteroides and Gram-positive Firmicutes dominate the gut of healthy individuals [11]. The gut microbiota forms a long-term, dynamically balanced symbiotic relationship with the host, which plays an important role in human immunity and metabolism. The association between gut microbiota imbalance and many neurodegenerative diseases supports the importance of the gut microbiota in tipping the balance between health and disease [12]. Changes in the gut microbiota can cause inflammation and affect the immune system [13], and these effects, in turn, have an impact on the CNS [14,15]. Effects on the immune system are thought to be the primary mechanism by which the microbiota affects the development of NMOSD. Our purpose is to summarize research evidence on the involvement of the gut microbiota in the pathogenesis of NMOSD and its potential regulatory mechanisms.

## 2. Development and Function of the Microbiota

### 2.1. Developmental Processes and Factors Affecting the Gut Microbiota

The gut microbiota is largely acquired from the mother at birth and continues to multiply through feeding and contact with the external environment [16]. The infant microbiota evolves rapidly during the first 2–3 years of life, and its composition is subject to influence from various factors such as the mode of delivery, nutritional source, antibiotic exposure, and geographic location [17]. And although the microbiota stabilizes upon reaching childhood, its composition is still thought to change in response to environmental changes [18].

Diet is undoubtedly a major factor influencing changes in the gut microbiota [19]. Diet determines which bacteria will thrive in the gut, and the overall characteristics of the diet [20], as well as individual components such as carbohydrates [21], proteins, fats, fibers, and vitamins, affect the composition of the microbiota, and dietary modifications can result in detectable changes in certain bacterial species within 24 h [22,23].

In addition, aging may be another key factor in changes in the gut microbiota, which is particularly important given the age-specific nature of many neurodegenerative diseases [24]. Among the elderly, poor diet is associated with lower microbiota diversity, inflammation, and disability [20]. In elders with other physical comorbidities, the use of medications, or limitations in terms of nutritional intake due to dysphagia or gastrointestinal dysfunction, may also alter the composition of the gut microbiota [25]. It is worth noting that some other social individual factors, such as cigarette smoking, have also been associated with inflammatory bowel disease [26]. Psychological stress and depression can also remodel the composition of the gut microbiota through stress hormones, inflammation, and autonomic changes [27]. These effects should also be taken into account when assessing microbial patterns associated with specific neurodegenerative diseases.

### 2.2. The Role of the Gut Microbiota in Health

The gut microbiota plays a pivotal role in the development and maturation of the mucosal immune system [28], maintains the integrity of the intestinal barrier [29], regulates intestinal neuromuscular function [30], and performs many key metabolic functions [31]. The gut microbiota is also capable of producing a variety of antimicrobial and bacteriostatic substances [32]. Microbial molecules produced or transformed by microorganisms are major players in the dialog with immune cells [33]. Specifically, the gut microbiota metabolizes through fermentation [34] and modifies host-derived metabolites to produce certain vitamins, fatty acids, amino acids, and polyamines essential for immune regulation [35]. In addition, the gut microbiota catalyzes the metabolism of drugs, carcinogens, and hormones, reduces histamine synthesis, and acts as a detoxifier.

Gut microbiota chemical signaling not only triggers the expression of enzymes, receptors, and transcription factors but also releases bioactive substances to regulate physiology and health [36]. Dysbiosis of the gut microbiota can lead to alterations in microbiota-derived components and metabolites, which can lead to dysregulation of the immune system and induce or participate in disease [37]. The interactions, interdependence, and mutual exclusion of different microbiota result in the gut microbiota exhibiting great diversity and variability at each stage of health and disease [38,39].

### 2.3. The Role of Gut Microbiota in Neurodevelopment

Recently, the effects of the gut microbiota on physiological systems have been expanded to include effects on the nervous system. During development, the nervous system is assembled and shaped by a series of temporally regulated developmental processes that form critical functional neural circuits. These processes began in utero and were subsequently refined and modified during early postnatal development [40]. As pregnancy advances, the diversity of the human gut microbiota increases, leading to a significant difference between the composition of the gut microbiota between late and early pregnancy [41]. Interestingly, these changes occur at the same time as neurodevelopmental plasticity, and they share a similar critical developmental window of sensitivity to injury [42]. Any disturbance during this period may have profound structural and functional effects on brain development and increase the risk of neurodevelopmental disorders [43]. Some microbiota-deficient mice exhibit increased BBB permeability, as well as altered gene expression related to neurotransmission, neuronal plasticity, metabolism, and morphology in the amygdala and hippocampus [44,45]. Other preclinical studies using germ-free mice have emphasized the ability of the microbiota to influence neurodevelopment early in life [46,47,48].

In addition, gut microbiota metabolites are important for normal neuronal function. Symptoms of metabolic syndrome, including increased adiposity, inflammation, and impaired glucose tolerance in germ-free mice transplanted with human microbiota during late pregnancy, have been shown to be potentially related to key gut microbial metabolites short-chain fatty acids [49,50]. The role of commensal microbes in the production of short-chain fatty acids and aryl hydrocarbon ligands, which may alter the development and function of glial cells within the CNS, has also been emphasized. Neuroendocrine mediators produced through the hypothalamic–pituitary–adrenal axis (HPA) can influence intestinal permeability, immune cell activation, and the composition of the gut microbiota [51]. For example, 5-hydroxytryptamine (serotonin) was produced primarily by enterochromaffin cells in the gut, and its production was largely influenced by commensal bacteria [52,53].

Although there appears to be a critical window for the development of the nervous system and microbiota, the precise underlying mechanisms of these effects remain to be elucidated. Uncovering the mechanisms that trigger these will improve our understanding of the etiology of neurodegenerative diseases and identify new opportunities for developing therapeutic interventions.

## 3. The Pathophysiology of NMOSD

### 3.1. Neuroimmunological Perspective

Neuroimmunology is dedicated to the study of the complex relationships and interactions between the CNS and the immune system to maintain homeostasis in the body and respond to infection, injury, or disease [54]. Traditionally, the immune system was thought to be autonomous, while the brain was protected by the BBB. However, findings in recent decades suggest an interdependent communication mechanism between the nervous and immune systems. In addition, both immune cells and neurons interact directly or indirectly with microorganisms. This interaction between the nervous system, the immune system, and the microbiota has been shown to play a role in controlling the overall response of the host [55].

The autoimmune response in NMOSD is driven by the binding of pathogenic AQP4-Ab to astrocytes surrounding endothelial cells [56]. Antibodies to NMO-IgG target conformational determinants exposed to the extracellular loop of AQP4, and AQP4-Ab, together with complement, can damage or destroy astrocytes [57]. CNS lesions in patients with NMOSD are characterized by rosette-like deposits of IgG, IgM, and complement, most notably in the perivascular area [58]. Experimental data suggested that AQP4-Ab induced interleukin 6 (IL-6) production by astrocytes and that IL-6 signaling decreases BBB function [59], thereby permitting autoimmune responses to occur within the CNS. AQP4-Ab crosses the BBB and binds to the extracellular structural domain of the AQP4 receptor, which triggers activation of the classical complement cascade, followed by the infiltration of granulocytes, macrophages/microglia, and T cells infiltration [58]. Eventual damage to astrocytes leads to loss of support for peripheral cells such as oligodendrocytes and neurons with oligodendrocyte damage and demyelination, neuronal loss, and neurodegeneration [60]. Demyelination affects gray and white matter, sometimes with necrosis and cavities, as well as vascular thickening and hyalinization [61]. Preservation or secondary loss of neurons will depend on the severity of the disease.

#### 3.1.1. The Role of T Cells in NMOSD

AQP4-Ab are T-cell-dependent IgG1 [62]. The humoral immune response against AQP4-Ab triggers specific CD4^+^ T cells via major histocompatibility complex (MHC) II [4,63]. CD4^+^ T cells from NMOSD patients exhibit T-helper cell (Th)17 polarization, which is partially mediated by an increased production of the pro-inflammatory cytokine IL-6 [9]. The signature cytokine of Th17, IL-17, has been found to be increased in the cerebrospinal fluid and blood of NMO patients [64,65,66]. IL-17 impairs BBB integrity, promotes endothelial activation, and stimulates neutrophil migration across the endothelium [67]. In addition, several studies have reported an elevation in the expression level of IL-6 in the cerebrospinal fluid and blood of NMO patients [68]; it could increase the survival of plasmacytoid cells producing anti-AQP4-IgG and be involved in the development of Th17 cells [69].

Additionally, Th17-related markers have been shown to correlate with NMOSD disease activity and severity. IL-6 and IL-21 released by CD4^+^ T cells from NMO patients have been directly linked to neurologic dysfunction [70], and the in vivo level of IL-6 was higher at a 2-year follow-up in relapsed NMO patients [71].

In addition to CD4^+^ T cells, CD8^+^ T cells have also been found to be involved in the immune cascade response in the development of NMOSD [61]. It was found that the frequency of naïve CD8^+^ T cells in the NMOSD circulatory system was significantly reduced, whereas the frequency of effector/memory CD8^+^ T cells was increased [72], suggesting that the CD8^+^ T cell subset was associated with the pathogenesis of NMOSD. Z. Shi et al. also found that NMOSD patients not only had a higher proportion of IFNc^+^CD8^+^ T cells but also a significantly higher number of TNFa^+^CD8^+^ T cells by detecting pro-inflammatory cytokine secretion [72]. This was thought to be related with the severity of NMOSD [73,74]. However, whether these pro-inflammatory cytokines produced by CD8^+^ T cells can serve as potential biomarkers for monitoring the development of NMOSD needs to be further investigated.

Recent studies also found a correlation between the expansion of circulating T follicular helper (Tfh) cells and disease activity in NMOSD patients [75,76,77]. Yick et al. demonstrated that the inhibition of Tfh cell expansion reduced Th1, Th17, memory B cell, and plasma cell responses and improved dyskinesia and NMOSD-like pathology in an AQP4 autoimmune mouse model [78]. These observations demonstrated the potential role of AQP4-specific T cells as drivers of adaptive humoral and cellular immune responses in the pathogenesis of NMO.

#### 3.1.2. The Role of B Cells and Plasma Cells in NMOSD

B cells, plasma cells, and antibody B cells can perform a variety of normal functions, and when these functions are dysregulated, they may influence NMOSD disease activity. There are several hypothesized mechanisms by which B cells play a role in NMOSD immunopathology: abnormalities in B cell tolerance checkpoints, AQP4 autoantibody production, increased pro-inflammatory B cell and plasma cell activity, diminished B cell regulatory function, and loss of B cell unresponsiveness [79].

During early B cell development, immunoglobulins generate B cell receptors (BCRs), autoreactive B cells, and cells that contain an initial pool of non-autoreactive cells. The autoreactive B cell pool is considered to be a reservoir of derived disease-associated autoantibodies. Most autoreactive B cells can be eliminated by two separate steps of the tolerance mechanism [80], and defects in the B cell tolerance checkpoint may lead to AQP4-Ab production. Chihara et al. have identified a population of CD19intCD27high CD38high CD180 B cells that are selectively increased in the peripheral blood of NMOSD patients, which secreted AQP4-Ab after IL-6 stimulation [81]. The pathogenic role of AQP4-Ab highlights the importance of B cells in NMOSD.

It has also been suggested that the contribution of B cells to the pathogenesis of NMOSD may go beyond the production of AQP4-Ab. B cells may act as potent antigen-presenting cells and participate in the development of Tfh cells [82,83]. B cells may also exacerbate disease activity by producing IL-6 to promote pathogenic Th17 differentiation [84]. The presence of AQP4-specific B cells may promote T cell responses to AQP4, leading to tissue damage. Thus, cooperation between AQP4-specific B cells and AQP4-specific T cells may be particularly important in NMOSD production.

Stimulated B cells leaving the germinal center may differentiate into plasmablasts and plasma cells. Plasma cells secrete factors such as IL-17, tumor necrosis factor a (TNF-a)/nitrous oxide (NO), and granulocyte macrophage colony-stimulating factor (GM-CSF) [85,86], which promote the infiltration of CNS neutrophils and macrophages. Anti-inflammatory plasma cells (Pregs) may inhibit pro-inflammatory T cell function through the production of IL-10, thereby limiting the immune response [87]. In addition, cytokines produced by various plasma cell subpopulations modify adaptive and innate immune responses by modulating humoral immunity and altering interactions with the commensal microbiota [88].

Another mechanism that contributes to the suppression of humoral autoimmunity, B cell unresponsiveness, has been relatively unexplored in detail in NMOSD [89]. During disease relapse, unresponsive escape may increase the frequency of circulating AQP4-IgG1 plasmablasts, which in turn increases AQP4-IgG titers [90]. These collective findings provide new insights into the important role of B cells in NMOSD immunopathology.

### 3.2. Endogenous and Exogenous Factors

In addition to neuroimmune factors, there was substantial evidence that NMOSD was associated with a complex interplay between genetic susceptibility and environmental factors. Ethnic groups and geographic location may influence the prevalence, age of onset, and severity of attacks. A study of Asian patients showed that the prevalence was significantly higher among Chinese than Malays patients. Additionally, the prevalence of NMOSD appears to be higher among people of East Asian origin (Chinese and Japanese) than in other Asian groups [91]. An international cohort study [92] showed that the age of onset of the disease was younger in patients of Asian and African descent than in white patients, and that the frequency of severe exacerbations was significantly higher in patients of African descent than in Asian and white patients.

Furthermore, human leukocyte antigens HLA-A*01 and HLA-B*08 have shown to be identified as having associations with NMOSD [93]. In a cohort study, HLADRB1*08:02 and HLA-DPB1*05:01 were found to be susceptible alleles, whereas HLA-DRB1*09:01 was found to be protective [94]. Similarly, the association of HLA with NMOSD has been affected by ethnicity and geography. HLADRB1*03, HLA-DPB1*0501, and HLA-DPB3 have been associated with increased susceptibility to NMOSD in Asian, Caribbean Afro-descendant, and Brazilian Afro-descendant populations [95], while HLA-DQA1*05:03 has been significantly associated with Japanese patients [96].

Environmental factors relevant to influencing the onset or recurrence of NMOSD also include infectious diseases. Some of the infectious agents associated with NMOSD are more prevalent in Asia and Latin America than in Europe and North America, with a concomitant higher prevalence in that geographic region. A number of studies have shown that 20–30% of patients with recurrent NMOSD have a history of viral infection or infectious disease prior to clinical exacerbation [97,98]. Interestingly, low levels of vitamin D and insufficient exposure to ultraviolet light may also be associated with an increased risk of NMOSD. A cross-sectional study found increased sunlight exposure and serum osteodiol (a metabolite of vitamin D) in AQP4-Ab-negative NMOSD patients compared to AQP4-Ab-positive patients [99].

New evidence suggested that the gut microbiota plays an integral role in the pathogenicity of NMOSD. A defective gut barrier links gut bacteria to immune activation, producing pro-inflammatory cytokines that lead to a systemic inflammatory response, which in turn compromises the BBB and promotes neuroinflammation, ultimately leading to neurological damage and degeneration [100]. Ecological dysregulation of the gastrointestinal tract may promote pro-inflammatory T cell polarization. Conversely, cellular and humoral immunity can influence gut microbial community composition [101,102]. Host–microbiota interactions involving the intestinal epithelial/immune system have been extensively studied, and in the following chapters, we will explore the important link between the gut microbiota and NMOSD.

## 4. The Gut Microbiota Associated with NMOSD

### 4.1. Clostridium perfringens

*Clostridium perfringens* is ubiquitous in the normal gut and is a diverse species that has been recognized as a pathogen in a variety of contexts [103]. Notably, *Clostridium perfringens* is the species most significantly enriched in NMOSD patients [104]. Studies have shown an increase in the number of harmful *Clostridium perfringens* species in NMO patients [9]. In addition, a study by Pandit et al. of 39 Indian NMOSD patients showed [105] that the prevalence of *Clostridium boltae* was significantly higher in the fecal samples of AQP4-positive patients compared to AQP4-negative patients. And in AQP4-positive NMOSD patients, *Clostridium boltae* was associated with the expression of inflammatory genes associated with innate and acquired immunity, particularly plasma cell differentiation, B cell chemotaxis, and Th17 activation.

*Clostridium perfringens* was thought to be involved in the pathogenesis of NMOSD [63,104]. AQP4-specific T cell epitopes were found to contain residues with 90% homology to sequences within the adenosine triphosphate binding cassette transporter permease (ABC-TP) expressed by *Clostridium perfringens* [106]. Th17 cells from these patients recognized immunodominant AQP4 epitopes and proliferated in response to the corresponding ABC-TP peptide of *Clostridium perfringens*. A commensal anaerobic Gram-positive spore-forming bacteria closely related to the genus *Clostridium* was found to be associated with the differentiation of pro-inflammatory Th17 cells in mice [107]. Thus, *Clostridium* may alter the balance between pro-inflammatory and anti-inflammatory T cell subsets in human disease. In addition, epsilon toxin from *Clostridium perfringens* was able to cross the BBB and damage neurons, astrocytes, and oligodendrocytes [108]. Epsilon toxin has been shown to damage the optic nerve in vivo [109].

### 4.2. Streptococcus

*Streptococcus* is a type of Gram-positive bacteria that is widely distributed in the normal flora of humans and animals [110]. *Streptococcus* and streptococcal toxins have been reported to disrupt cytoplasmic integrity, destroy mucosal skin resistance, and cause inflammation [111]. Although the role of *streptococcus* in the pathogenesis of NMOSD has not been identified, more *streptococcus* in the colonic mucosa may disrupt epithelial cell integrity. A study of 84 Chinese patients with NMOSD showed that *streptococcus* was significantly increased in NMOSD and positively correlated with disease severity. The use of immunosuppressants led to a decrease in the number of *streptococci*, suggesting that *streptococcus* may play an important role in the pathogenesis of NMOSD [112]. The same group of studies from China showed that although there was an overall decrease in the diversity of bacterial flora in samples taken from six NMOSD patients, a significant amount of *streptococcus* and *Granulicatella* sp. were detected [113]. Another study from China supported these results with an overrepresentation of pathogenic bacteria such as *streptococci* and *flavonoids* in stool samples from NMOSD patients [114].

### 4.3. Other Microbiotas

Cui et al. showed that the gut microbiota of NMOSD patients had higher levels of *Streptococcus*, *Alistipes*, *Haemophilus*, *Veillonella*, *Butyricimonas*, and *Rothia*, and *Clostridium*, *Parabacteroides*, *Oxalobacter*, and *Burkholderia* were lower in abundance [113]. Notably, *Parabacteroides*, *Veillonella*, *Lactobacillus*, *Oscillibacter*, and *Streptococcus* were positively correlated with both disability and recurrence rates [115]. Colonization of the gut microbiota in germ-free mice showed that the selective inhibition of growth of *Bifidobacterium* and *Lactobacillus* reduced the number of Th17 cells [116].

Another cross-sectional study [114] showed a significant increase in *Flavonifractor* and *Streptococcus* among pathogenic bacteria in the NMOSD group. In addition, several intestinal commensals were detected in significantly lower abundance in NMOSD patients, including *Faecalibacterium*, *Lachnospiracea_incertae_sedis*, *Prevotella*, *Blautia*, *Roseburia*, *Romboutsia Coprococcus*, and *Fusicatenibacter*. An overview of the articles discussed in this section is presented in Table 1.

## 5. Immunological Mechanisms of Gut Microbiota in the Pathogenesis of NMOSD

### 5.1. Communication Mechanisms

The concept of the “enteric neuroimmune cell unit” has been developed to help understand the interactions between the nervous system and the immune system; it is an internal sensory organ responsible for protecting and maintaining the integrity and function of the intestinal tract [51]. This intestinal neuroimmune system plays an important role in providing innate defense and memory responses against certain pathogens. This system develops through colonization of the gut by commensal microorganisms, resulting in mutual signaling between neuron-glia and tissue-resident immune cells, stimulating the maturation of the enteric neuroimmune system [51].

Communication between the gut microbiota and the nervous system may be driven by the gut–brain axis (GBA) pathway [117]. More recently, due to the recognition of the importance of the microbiota in regulating health, the GBA has been expanded to include the microbiota–gut–brain axis [118], which represents a complex network of communication between the gut microbiota and the brain in the regulation of immune, gastrointestinal, and CNS functions [119].

Gut microbiota was transmitted to the brain via multiple different signaling pathways in the GBA, including neural, endocrine, HPA, and immune signaling [120]. Conversely, signals from the CNS and neuroendocrine system may monitor and regulate changes in the gut microbiota in response to environmental changes [121]. Enteroendocrine cells were thought to be a key cell type connecting the gut microbiota to the nervous system, because they monitor nutrient availability and the presence of non-nutrient chemicals, toxins, and microbes [122]. This communication between the gut microbiota and the nervous system was facilitated by the synthesis and release of many molecules, including neuropeptides, neurotransmitters, cytokines, and microbial metabolites [123,124]. They are important mediators between the nervous system and effector tissues and are very important in multimodal neuronal communication [125]. What has been learned about the interactions between the gut microbiota and the neuroimmune system has revealed their importance to overall health.

### 5.2. Roles of Gut Microbiota-Derived Metabolites in Immune Responses

**Figure 1:**
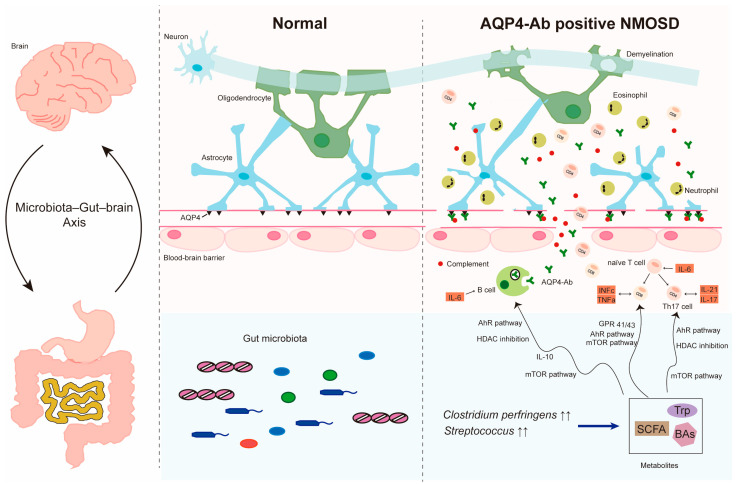
Pathogenesis of NMOSD and the role of gut microbiota. It is thought that an impaired immune system may promote naive T cell transformation into Th 17 cell and stimulate B cell differentiation to plasmablasts, then to plasma cells producing AQP4-IgG autoantibody. Interleukin 6 (IL-6) is a key factor in AQP4-related NMOSD pathophysiology. Besides IL-6, Th17 cells differentiation may be induced by IL-17 and IL-21(shown in small black arrows). A leaky BBB contributes to the migration of AQP4-IgG from the periphery into the CNS. AQP4-IgG bind to AQP4 and activate the complement cascade. Activated complement leads to leukocyte infiltration, particularly neutrophil and eosinophil, and results in astrocyte damage and demyelination. The gut microbiota has also been found to be involved in the pathogenesis of NMOSD. Metabolites produced by gut microbiota (shown in deep blue arrows) can pass through the damaged BBB and regulate T cells and B cells through multiple pathways (shown in long black arrows). Communication between the immune gut microbiota and the CNS may be driven by the microbiota–gut–brain axis.

Although recent studies have laid the groundwork in determining the role of the gut microbiota in neuroimmune regulation, the molecular signaling mechanisms by which the gut microbiota influences host neuroimmune homeostasis remain unclear. Several studies have emphasized the impact of metabolites produced by specific microbes in the gut flora, such as short-chain fatty acids (SCFAs), tryptophan (Trp), and bile acids (BAs) on immune cell activation and differentiation [126,127]. Investigating microbiota-neuroimmune interactions will reveal the fundamental relationship between the gut and the brain and inform the development of novel therapies for the treatment of various neurological disorders (Figure 1).

#### 5.2.1. SCFAs

A significant reduction in SCFAs was observed in the feces of patients with NMOSD, and acetate and butyrate were negatively correlated with disease severity, suggesting that reduction in SCFA levels may be another important feature of patients with NMOSD [112]. SCFAs are a key factor in the immune response of the mucosa [128]. Undigested carbohydrates can be fermented by the gut microbiota to SCFAs, which regulate the function of different immune cells by inhibiting histone deacetylase (HDAC) 40 and/or activating G protein-coupled receptors (GPR) [129]. Colonization of the gut by SCFA-producing bacteria also reduces BBB permeability, suggesting a key regulatory role of SCFAs in BBB development and maintenance [130].

CD4^+^ T cells. SCFAs exhibit significant effects on CD4^+^ T cells and have been reported to induce Th17 differentiation through the inhibition of HDAC and regulation of the mTOR pathway [131]. In addition, the production of IL-10 during SCFA-induced Th17 cell differentiation was also dependent on the inhibition of HDAC activity [132,133]. SCFAs are an important metabolite in CD4^+^ T cells for the inhibition of HDAC activity [134]. Another SCFA produced by a subdominant microbiota, SCFA–valeric acid, can stimulate the production of IL-10 by providing additional acetyl-coenzyme A (CoA) to histone acetyltransferases and enhancing glycolysis and mTOR activity [133]. Recent studies have also shown that the effect of SCFAs on T cell metabolism depends on the inflammatory environment, suggesting another layer of complexity [135].

CD8^+^ T cells. Research on how gut microbiota-derived metabolites affect CD8^+^ T cells is still in its early stages. SCFAs have been shown to enhance CD8^+^ T cell function by altering cellular metabolism [136]. Increased systemic acetate levels following bacterial infection promote rapid recall responses in memory CD8^+^ T cells [137]. Acetate also promotes IFN-γ production in CD8^+^ T cells by modulating mTOR activity [138].

In addition, SCFAs stimulate oxidative phosphorylation (OXPHOS) and mitochondrial mass and their glycolytic capacity in CD8^+^ T cells, and part of these changes depend on the activation of GPR41 [139]. GPR41 was essential in the SCFA-mediated expansion of CD8^+^ T cell function [136]. A recent study reported that butyrate enhanced memory CD8 ^+^ T cell responses dependent on GPR41 and GPR43 [139]. In contrast, butyrate promotes IFN-γ and granzyme B in CD8^+^ T cells due to the inhibition of HDAC activity rather than through the activation of GPR41 and GPR43 [140].

B cells. In addition, SCFAs can regulate the expression of genes associated with B cell differentiation through HDAC inhibition [141]. SCFAs promote plasma cell differentiation and antibody production by enhancing cytosolic acetyl-CoA levels in B cells, leading to higher mitochondrial energy production, fatty acid synthesis, and mTOR-mediated glycolysis [141].

Recent studies have shown that branching SCFAs such as isobutyric or isovaleric acid can also modulate B cell function. Lack of branching SCFA production in genetically manipulated germ-free mice of Clostridium perfringens resulted in an increased frequency of IgA^+^ plasma cells in the small intestine, as well as increased levels of IgA bound to the surface of innate immune cells [142]. However, the mechanisms underlying these effects are not clear. SCFAs can also regulate B cell IgA production by interacting with T cells. Given that IL-10 was a key cytokine for B cell differentiation and antibody maturation [143], SCFAs also indirectly affect B cell function by inducing IL-10 in CD4^+^ T cells [144]. It has also been reported that the effect of SCFAs on B cell responses was dose-dependent, with lower doses of SCFAs moderately enhancing antibody production, while higher doses of SCFAs inhibit antibody production [145].

#### 5.2.2. Trp Metabolites

Gut microbiota-derived tryptophan catabolic metabolites are mainly supplied by dietary proteins. The tryptophan product indole-3-acid-acetic (IAA) can reduce Th17 cell numbers by activating the aryl hydrocarbon receptor (AhR) pathway and downregulating retinoic acid receptor-related orphan receptor gamma t (RORγt) and signal transducer and activator of transcription 3 (STAT3) [146]. In contrast, the tryptophan product 6-formylindole(3,2-b) carbazole (FICZ) promotes the transformation of T cells into Th17 cells [147]. Kynurenine (Kyn) can mediate the immunosuppressive response by interacting with the ligand-activated AhR to upregulate PD-1 expression in CD8^+^ T cells [148]. 3-Hydroxyanthranilic acid (3-HAA) in the Kyn pathway induces immunosuppression by inducing the apoptosis of T cells through glutathione depletion [149]. Spermine, a polyamine, may regulate T cell responses by inhibiting the secretion of the Th1 cytokine IFN-γ by splenocytes [150]. Ascorbic acid, a novel microbial metabolite, inhibits T effector cells and suppresses T cell activation [151].

There are also several reports on the role of microbiota-derived tryptophan in B cells. The activation of tryptophan metabolism has been associated with the differentiation of B cells into antibody-secreting cells [152]. Tryptophan catabolic metabolites may regulate B cell development, differentiation, cytokine production, and activity through AhR signaling [153,154,155]. Studies have shown that butyrate administration increased tryptophan metabolism bacteria in mice in an AhR-dependent manner to promote IL-10-producing regulatory B cells [156]. However, how microbiota-derived tryptophan directly regulates B cell IL-10 production as well as antibody production remains unclear.

#### 5.2.3. BAs Metabolites

BA also has an important effect on T cells; BA metabolites (BA) control the differentiation of Th17 and Treg cells [157]. 3-oxoLCA, a derivative of lithocholic acid (LCA), can inhibit Th17 cell differentiation by blocking the function of RORγt [158]. RORγt was selectively expressed by Th17 and innate lymphocyte cell group 3 (ILC3) and was critical for the differentiation of these cells in chronic inflammation and autoimmune diseases [159]. In contrast, another derivative of LCA, isoalloLCA, promotes Treg cell differentiation by stimulating the production of OXPHOS and mROS and increasing the level of histone (H3K27) acetylation in the Foxp3 promoter [157].

BA metabolites also disrupt intracellular calcium homeostasis, which is critical for the nuclear factor for activating T cells (NFAT) signaling and T cell activation [160]. 24-Nourrsodeoxycholic acid (NorUDCA) alters the immunometabolism of CD8^+^ T cells and attenuates hepatic inflammation [161]. It has potent immunomodulatory effects on CD8^+^ T cells, affecting targets of lymphoblastogenesis, expansion, glycolysis, and target of rapamycin complex 1 (mTORC1) signaling. The BA metabolite-mediated activation of the VDR reduces sustained B-lymphocyte proliferation, induces the apoptosis of activated B cells, and inhibits Ig production. In addition, BA receptor VDR activation reduces the sustained proliferation of T lymphocytes [162,163,164]. An overview of the articles discussed in this section is presented in Table 2.

Several studies have also shown that the gut microbiota was necessary to trigger myelin autoantigen recognition to exacerbate autoimmune demyelination [165,166]. In astrocytes, AhR signaling acts synergistically with the microbiota and the neuroimmune landscape to regulate neuroinflammatory responses. AhR ligand synthesis requires gut microbe-mediated dietary metabolism of Trp to indole. Its activation suppresses NF-κB, the main regulator of pro-inflammatory responses [127]. Elucidating the molecular basis of microbiota–astrocyte interactions will provide additional information for assessing the ability of microbiota-based interventions in neurological disorders.

## 6. Fecal Microbiota Transplantation (FMT) in NMOSD

The above evidence suggests that the gut microbiota may play a role in the pathophysiology and pathogenesis of NMOSD. Therefore, changing the composition of gut bacteria may affect the course of NMOSD. Whether it is possible to change the gut microbiota of NMOSD patients through FMT, thereby benefiting NMOSD patients, deserves to be the target of our further exploration.

FMT refers to the transplantation of the gut microbiota from the feces of healthy individuals into the gastrointestinal tract of patients to repair changes in the gut microbiota’s structure in the disease to a certain extent to rebuild the recipient’s intestinal microecology [167]. An increasing number of studies have reported the use of FMT to influence the immune system and ameliorate immune-mediated diseases [167,168]. FMT plays a vital role in the treatment of autoimmune enteritis, hepatitis B, chronic non-alcoholic cirrhosis, epilepsy, autism spectrum disorder, etc. [169]. FMT positively affects the composition and function of the microbiota by increasing the levels of SCFAs. Therefore, it is theoretically feasible to apply FMT to diseases with Th17/Treg cell imbalance and may be an effective therapy for the treatment of human neuroimmune diseases [11].

However, the current level of evidence from clinical studies is insufficient. To date, research on FMT for the treatment of many neuroimmune diseases has been limited to animal models. FMT can change the microbial community and immune adaptability of laboratory mice [170]. Although this result can be used to predict human immune responses, it is unclear whether the results obtained in animal experiments can be directly applied to human patients. In most cases, the causative pathogen is unlikely to be a single microorganism. Considering the differences in gut microbiota between individuals, we ask whether treatment strategies that can carry out personalized medicine for patients require in-depth consideration. In the pathogenesis of NMOSD, microbial composition only participates in certain steps in the pathogenic pathway as a control factor, and FMT may or may not necessarily have a therapeutic effect. In addition, as a biological treatment method, FMT also has certain side effects. The most common manifestations are constipation, diarrhea, abdominal pain, and transient low-grade fever. These side effects are the body’s natural response to the introduction of live microorganisms and their metabolites and generally resolve within days to weeks [171].

While current research sheds light on the association between individual bacterial groups and NMOSD, our understanding of the gut microbiota in NMOSD is only scratching the surface. An important question that should be considered is whether these changes precede the development of NMOSD or are secondary to the disease itself. Most studies of the gut microbiota in NMOSD are often descriptive, and studies investigating how the gut microbiota directly affects NMOSD remain sparse. Therefore, more research is needed to distinguish correlation and causation between the gut microbiota and NMOSD. At the same time, due to the potential impact of immunosuppressive therapy on the gut microbiota, future studies should include large numbers of untreated NMOSD patients.

## 7. Conclusions

The microbiota–gut–brain axis is the main pathway through which the gut microbiota affects the CNS. The microbiota is critical to the maturation and function of immune cells and glia. Differences in many bacterial taxa found in NMOSD patients supported the importance of the gut microbiota in the pathophysiology of NMOSD, such as *Clostridium perfringens* and *Streptococcus*. The gut microbiota-derived metabolites SCFAs, BAs, and Trp are also particularly important in the metabolic network of immune cells and gut microbiota. Deciphering all the mechanisms of the microbiota effects on immunometabolism is extremely challenging.

The role of the gut microbiome in the pathophysiology of NMOSD is very important. It can serve as a biomarker for disease onset and progression and as a potential disease-modifying therapy. In recent decades, significant progress has been made in immunology and microbiology using microbiota, germ-free animals, and next-generation sequencing [172] to reveal the correlation between the gut microbiota and the immune system. Based on this, mouse models of FMT in humans and wild-type mice are expected to improve the success rate of preclinical studies. Microbial therapies by improving the intestinal microbiota are expected to open up new avenues for the treatment and prevention of certain neuroimmune diseases.

## Figures and Tables

**Table 1:** An overview of studies investigating the role of gut microbiota in NMOSD.

Samples	Number of NMOSD Patients	Gut Microbiota	Results
Peripheral blood T cells	15	*Clostridium perfringens*	1. AQP4-specific T cell epitopes were found to contain residues with 90% homology to sequences within the ABC-TP. 2. Th17 cells recognized immunodominant AQP4 epitopes and proliferated in response to the corresponding ABC-TP peptide.
Fecal samples	16	*Clostridium perfringens*	1. *Clostridium perfringens* is the species most significantly enriched in NMOSD patients.
Fecal and peripheral blood samples	39	*Clostridium boltae*	1. The prevalence of *Clostridium boltae* was significantly higher in AQP4-positive patients. 2. In AQP4-positive NMOSD patients, *Clostridium boltae* was associated with inflammatory genes.
Fecal samples	84	*Streptococcus*	1. *Streptococcus* was significantly increased and positively correlated with disease severity. 2. The use of immunosuppressants led to a decrease in the number of *streptococci*.
Sigmoid mucosal	6	*streptococcus* and *Granulicatella* sp.	1. An overall decrease in the diversity of bacterial flora. 2. Significant amount of *streptococcus* and *Granulicatella* sp. were still detected.
		*Streptococcus*, *Alistipes*, *Haemophilus*, *Veillonella*, *Butyricimonas*, and *Rothia*,	3. Higher levels.
		*Clostridium*, *Parabacteroides*, *Oxalobacter*, and *Burkholderia*	4. Lower abundance.
Fecal samples	20	*streptococci* and *flavonoids*	1. Overrepresentation.
		*Faecalibacterium*, *Lachnospiracea_incertae_sedis*, *Prevotella*, *Blautia*, *Roseburia*, *Romboutsia Coprococcus*, and *Fusicatenibacter*.	2. Lower abundance.
Fecal samples	22	*Parabacteroides*, *Veillonella*, *Lactobacillus*, *Oscillibacter*, and *Streptococcus*	1. Positively correlated with both disability and recurrence rates.

**Table 2:** The functions of the major gut microbiota-derived metabolites.

Metabolites	Functions	Mechanisms
SCFAs	Regulation of Th17 cells differentiation	Inhibition of HDAC and regulation of the mTOR pathway
	Stimulate the production of IL-10	Enhancing glycolysis and mTOR activity
	Acetate promotes IFN-γ production in CD8^+^ T cells	Modulating mTOR activity
	Butyrate promotes IFN-γ and granzyme B in CD8^+^ T cells	Inhibition of HDAC activity
	SCFAs stimulate OXPHOS, mitochondrial mass, and glycolytic capacity in CD8^+^ T cells	Activation of GPR41
	Regulate the expression of genes associated with B cell differentiation	Inhibition of HDAC activity
	Promotes plasma cell differentiation and antibody production	Enhancing cytosolic acetyl coenzyme A levels in B cells
	Regulate B cell IgA production	Interacting with T cells
Trp	IAA can reduce Th17 cell numbers	Activating the AhR pathway and downregulating RORγt and STAT3
	Kyn can mediate the immunosuppressive response in CD8^+^ T cells	Interacting with the ligand-activated AhR to upregulate PD-1 expression
	Spermine may regulate T cell responses	Inhibiting the secretion of the Th1 cytokine IFN-γ by splenocytes
	Regulate B cell development, differentiation, cytokine production, and activity	AhR signaling
	Butyrate promote IL-10-producing regulatory B cells	AhR-dependent manner
BAs	3-oxoLCA can inhibit Th17 cell differentiation	Blocking the function of RORγt
	IsoalloLCA promotes Treg cell differentiation	Stimulating the production of OXPHOS and mROS and increasing the level of H3K27 acetylation
	Reduces sustained B-lymphocyte proliferation, Induces apoptosis of activated B cells, inhibits Ig production, and reduces the sustained proliferation of T lymphocytes	Activation of the VDR

## Data Availability

Not applicable.
